# Global sagittal alignment of the spine, pelvis, lower limb after vertebral compression fracture and its effect on quality of life

**DOI:** 10.1186/s12891-021-04311-8

**Published:** 2021-05-24

**Authors:** Leo Tsz Ching Chau, Zongshan Hu, Koko Shaau Yiu Ko, Gene Chi Wai Man, Kwong Hang Yeung, Ying Yeung Law, Lawrence Chun Man Lau, Ronald Man Yeung Wong, Winnie Chiu Wing Chu, Jack Chun Yiu Cheng, Sheung Wai Law

**Affiliations:** 1 Department of Orthopaedics and Traumatology, Faculty of Medicine, The Chinese University of Hong Kong, Prince of Wales Hospital, Shatin, Hong Kong SAR China; 2 Spine Surgery, Drum Tower Hospital of Nanjing University Medical School, Nanjing, China; 3 Department of Imaging and Interventional Radiology, Faculty of Medicine, The Chinese University of Hong Kong, Prince of Wales Hospital, Shatin, Hong Kong SAR China

**Keywords:** Global sagittal alignment, Vertebral compression fracture, Biplanar radiographs, Quality of life, Lower limb

## Abstract

**Background:**

Vertebral compression fractures (VCFs) are the most common among all osteoporotic fractures. The body may compensate to the kyphosis from vertebral compression fractures with lordosis of the adjacent spinal segments, rotation of the pelvis, knee flexion and ankle dorsiflexion. However, the detailed degree of body compensation, especially the lower limb, remains uncertain. Herein, the aim of this study is to investigate the values of global sagittal alignments (GSA) parameters, including the spine, pelvis and lower limbs, in patients with and without VCFs, as well as to evaluate the effect of VCFs on various quality of life (QoL) parameters.

**Methods:**

A cross-sectional study was conducted from May 2015 to June 2018. A total of 142 patients with VCFs aged over 60 years old and 108 age-matched asymptomatic controls were recruited. Whole body sagittal alignment including thoracic kyphosis (TK), lumbar lordosis (LL), pelvic tilt (PT), pelvic incidence (PI), sagittal vertical axis (SVA), T1-pelvic angle (TPA), knee-flex angle (KA) and ankle-flex angle (AA) were measured. In addition, lower back pain and quality of life were assessed using self-reported questionnaires.

**Results:**

Compared to asymptomatic controls, patients with VCF showed significantly greater TK (33.4^o^ ± 16.4^o^ vs 28.4^o^ ± 11.4^o^; *p* < 0.01), PT (25.4^o^ ± 10.5^o^ vs 16.6^o^ ± 8.9^o^; *p* < 0.001), PI (54.6^o^ ± 11.8^o^ vs 45.8^o^ ± 12.0^o^; *p* < 0.001), SVA (49.1 mm ± 39.6 mm vs 31.5 mm ± 29.3 mm; *p* < 0.01), and TPA (28.6^o^ ± 10.8^o^ vs 14.8^o^ ± 8.6^o^; *p* < 0.001). Whereas for lower limb alignment, patients with VCF showed significantly higher KA (10.1^o^ ± 7.8^o^ vs 6.0^o^ ± 6.4^o^; *p* < 0.001) and AA (7.0^o^ ± 3.9^o^ vs 4.8^o^ ± 3.6^o^; *p* < 0.001) than controls. The number of VCF significantly correlated with lower limb alignments (KA and AA) and global sagittal balance (TPA). VCF patients showed poorer quality of life assessment scores in terms of SF-12 (30.0 ± 8.3 vs 72.4 ± 16.9; *p* < 0.001), ODI (37.8 ± 24.0 vs 18.7 ± 16.6; *p* < 0.001) and VAS (3.8 ± 2.8 vs 1.9 ± 2.2; *p* < 0.001).

**Conclusion:**

This is the first study to illustrate the abnormal lower limb alignment exhibited in patients with VCF. Patients with VCF showed an overall worse global sagittal alignment and decreased quality of life. Poorer global sagittal alignment of VCF patients also imply worse quality of life and more severe VCF.

## Background

Osteoporosis and its related complications have been increasing within the aging population. Vertebral compression fractures (VCF) are the most common among all osteoporotic fractures. The occurrence of this condition steadily increases as a person age, with an estimated 40% of women age 50 and older affected [1]. This manifestation occurs when the bony block or vertebral body in the spine collapses, which are associated with spinal deformity, chronic back pain, increased morbidity and mortality, and overall decline in quality of life (HRQoL) [2–4]. Importantly, people who have had one osteoporotic VCF are at five times the risk of sustaining a second VCF [5].

Sagittal spinal alignment has been reported to play an important role in the biomechanical adaptation of the spine in pathology. In response to VCFs, our body may compensate globally to the kyphosis by drastic change of the whole body sagittal alignment, with lordosis of the adjacent spinal segments, posterior tilting of the pelvis, hip extension, knee flexion and even ankle dorsiflexion, in order to maintain horizontal gaze and balance of the body [6]. These compensatory mechanisms aim to recreate an optimal alignment of the spine, with the objective of keeping the appropriate position of the gravity line, and the horizontal gaze. Previous studies have shown that VCF patients have higher thoracic kyphosis and lower lumbar lordosis [7]. Similarly, our team previously also investigated on the relationship between global sagittal alignment and VCF, and T1-pelvic angle was found to be increased in VCF patients [8]. Moreover, poor sagittal alignment of the spine in osteoporotic patients is reported to be an independent risk factor for subsequent VCF [9].

However, the detailed influence of lower limb compensation, including the contribution from the knee and ankle, in VCF patients remains unknown. Herein, the aim of this study is to investigate the values of global sagittal alignments (GSA), including lower limbs, in patients with and without VCFs, and to establish the relationships between GSA with other clinical and radiological parameter.

## Methods

### Study population

We prospectively recruited 142 elderly women aged over 60 who consulted for VCF in our institution from May 2015 to June 2018. The exclusion criteria for the current study are as follows: history of previous lower limb fracture, history of previous surgery of spine, pelvis or lower limb, rheumatic diseases and secondary osteoporosis (e.g., osteopenia with hyperparathyroidism, hyperthyroidism, chronic kidney disease, or osteomalacia). In addition, 108 age-matched subjects without VCF were recruited as asymptomatic controls from the whole of Hong Kong through designated advertisements, flyers and recruitment brochures. Subject aged over 60 years old and without VCF were subjected to confirmation on the absence of VCF and exclusion criteria by two orthopaedics surgeons (L.T.C.C. and S.W.L.). Written informed consent was obtained for all subjects before participating in this study. Ethical approval was obtained from the ethics review board of the joint NTEC-CUHK clinical research ethics committee. All study procedures were conducted in accordance with the guidelines approved by the ethics committee and the Declaration of Helsinki.

### Demographic data collection

Demographic characteristics of the subjects, including age, body height, and body weight, were recorded. Body mass index (BMI) was then determined. Quality of life assessment was completed for each subject by locally validated questionnaires: the Oswestry Disability Index (ODI), Short-form (SF)-12 and Visual Analogue Scale (VAS) [10–12].

### Low-dose Biplanar whole-body radiographic assessment

**Fig. 1:**
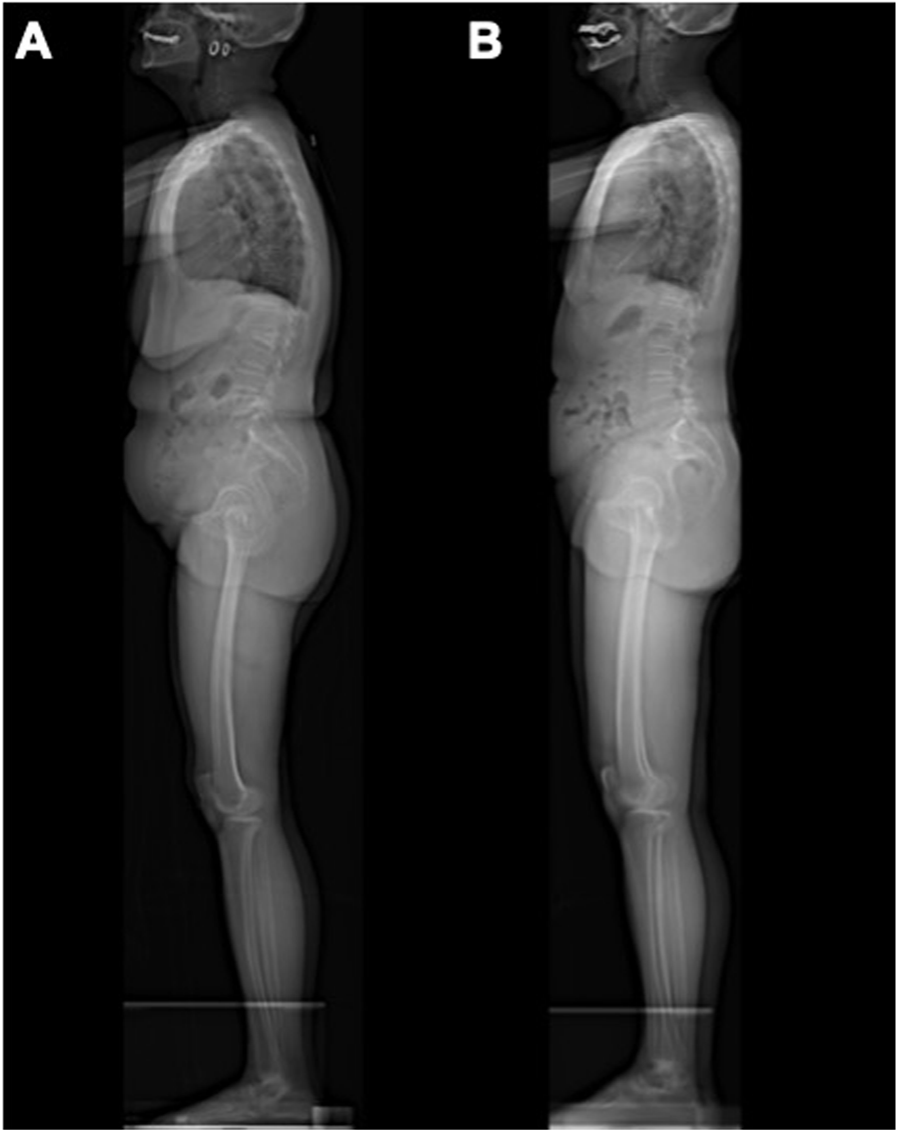
Representative radiographs of elderly adults recruited in this study. **a** 66 year-old female patient with VCF and **b** 66 year-old female patient without VCF in lateral views

**Fig. 2:**
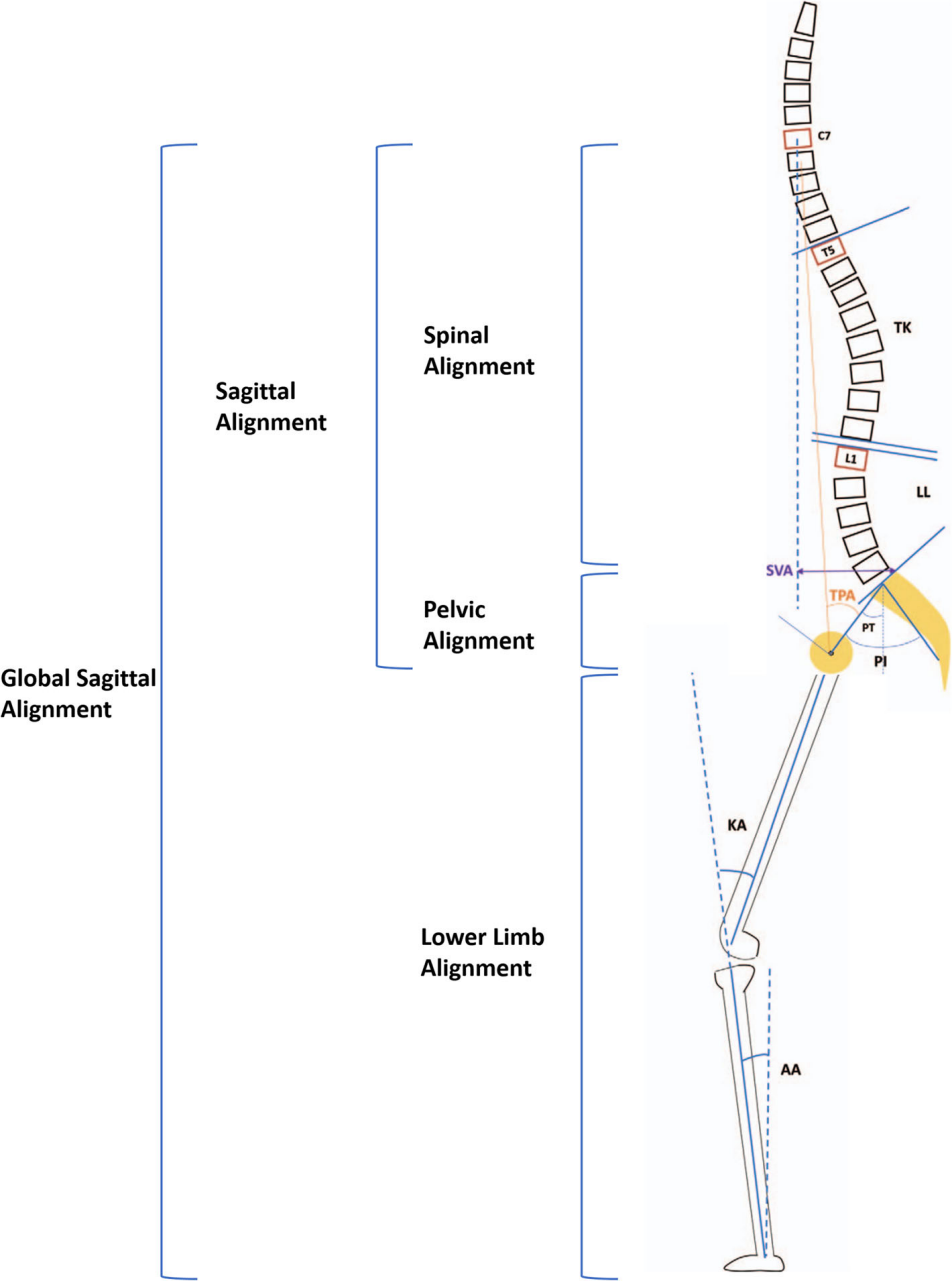
Illustration of measurements of spinal, pelvic, global and lower limb parameters in sagittal radiograph. AA indicates ankle flexion angle; KA indicates knee flexion angle; LL indicates lumbar lordosis; PI indicates pelvic incidence; PT indicates pelvic tilt; SVA indicates sagittal vertical axis; TK indicates thoracic kyphosis; TPA indicates T1-pelvic angle

All subjects underwent whole body biplanar stereographs (EOS imaging, Paris, France) with a standardized radiographic protocol by a team of experienced radiographer (Fig. 1). Subjects were instructed to stand in a comfortable position with hips and knees extended and with hands on a support [7]. EOS images were measured using validated software (Surgimap, Nemaris Inc., New York, NY) for sagittal parameters. Spinal parameters measured includes thoracic kyphosis (T5–12, TK) and lumbar lordosis (L1–S1, LL). Pelvic parameters measured includes pelvic incidence (PI) and pelvic tilt (PT). Global sagittal parameters measured includes sagittal vertical axis (SVA) and T1 pelvic angle (TPA: the angle between the line from the femoral head axis to the centroid of T1 and the line from the femoral head axis to the middle of the S1 superior endplate). Lower limb parameters evaluated includes knee flexion angle (KA: angle between the mechanical axis of the femur and the mechanical axis of the tibia) and ankle dorsiflexion angle (AA: angle between the mechanical axis of the tibia and the vertical axis) [13] (Fig. 2). In clinical practice, the outline of thoracic vertebra above T5 on the x-ray radiographs are often difficult to identify and mark in the sagittal plane, owing to the obstruction of rib cage and upper arm.

All the parameters were measured by two independent observers (L.T.C.C. and Z.H.). Intraobserver and interobserver variations were estimated by using intraclass correlation coefficient (ICC), which were graded using previously described semi-quantitative criteria: excellent (ICC ≥ 0.9), good (0.7 ≥ ICC < 0.9), acceptable (0.6 < ICC ≥ 0.7), poor (0.5 ≥ ICC < 0.6), or unpredictable (ICC < 0.5).

### Statistical analysis

Data were expressed as mean ± standard deviation. All analyses were conducted with the SPSS software (Version 25.0; SPSS, Chicago, IL, USA). Comparisons of means between variables were performed using independent Student t test. The correlations between variables were analyzed using the Pearson correlation coefficient. The intra- and inter-observer reliability were obtained as intraclass correlation coefficients (ICC). *P*-value less than 0.05 was considered statistically significant for all analyses.

## Results

### Patient demographic data

The mean age of the elderly subjects with vertebral compression fracture was 77.1 ± 8.4 years, including 132 females and 10 males. Whereas, 108 asymptomatic age-matched adults, including 64 females and 44 males, with mean age of 77.8 ± 8.1 years were compared. There is no significant difference on the age between VCF patients and asymptomatic controls. Patients with VCF are found to have lower height, weight and BMI when compared with controls (Table 1).

**Table 1:** Comparisons between the patients with and without vertebral fracture (VCF) in terms of baseline characteristics

Variables	Presence of VCF (***n*** = 142)	Absence of VCF (***n*** = 108)	***P*** value
Age (years)	77.1 ± 8.4	76.5 ± 8.4	0.567
Height (cm)	149.1 ± 6.9	156.4 ± 7.8	**< 0.001**
Weight (kg)	50.1 ± 8.4	59.2 ± 9.86	**< 0.001**
BMI (kg/m^2^)	22.5 ± 3.2	24.2 ± 3.2	**< 0.001**
SF12 (PCS)	30.0 ± 8.3	72.4 ± 16.9	**< 0.001**
ODI	37.8 ± 24.0	18.7 ± 16.6	**< 0.001**
VAS	3.8 ± 2.8	1.9 ± 2.2	**< 0.001**

*BMI* Body mass index, *SF-12* Short Form 12, *PCS* The physical component summary, *ODI* Oswestry disability index, *VAS* Visual analogue scale

Data expressed as mean ± SD

In addition, VCF patients has significantly lower SF-12 (*p* < 0.001), higher ODI (*p* < 0.001), and higher VAS (*p* < 0.001) than asymptomatic controls (Table 1). This indicates more pain and poorer quality of life in VCF patients than the age-matched asymptomatic controls.

### Reliability of the measurement between observers

The intra- and interobserver ICCs for estimating the whole-body sagittal parameters were from 0.83 to 0.96, suggesting good to excellent reliability of these measurements among the two observers (L.T.C.C. and Z.H.)

### Comparison on the GSA between subjects with and without VCF

The mean values and standard deviations of the radiographic parameters are showed in Table 2. Compared to asymptomatic controls, patients with VCF has significantly higher spinopelvic parameters in terms of TK (*p* < 0.01) PT (*p* < 0.001), and PI (*p* < 0.001). In addition, VCF patients also has significantly higher global parameters, in terms of SVA (*p* < 0.01) and TPA (*p* < 0.001), than the controls. The mean SVA in VCF patients and controls were 49.1 mm ± 39.6 mm and 31.5 mm ± 29.3 mm, respectively. Similarly, the mean TPA in VCF patients and controls were 28.6^o^ ± 10.8^o^ and 14.8^o^ ± 8.6^o^, respectively.

**Table 2:** Mean value of whole-body sagittal parameters between the patients with and without vertebral fracture (VCF)

Parameter	Presence of VCF (***n*** = 142)	Absence of VCF (***n*** = 108)	***P*** value
*Spinopelvic*			
Thoracic kyphosis (^o^)	33.4 ± 16.4	28.4 ± 11.4	**0.004**
Lumbar lordosis (^o^)	40.2 ± 18.0	40.8 ± 11.3	0.719
Pelvic tilt (^o^)	25.4 ± 10.5	16.6 ± 8.9	**< 0.001**
Pelvic incidence (^o^)	54.6 ± 11.8	45.8 ± 12.0	**< 0.001**
*Global*			
SVA (mm)	49.1 ± 39.6	31.5 ± 29.3	**0.003**
T1 pelvic angle (^o^)	28.6 ± 10.8	14.8 ± 8.6	**< 0.001**
*Lower-limb*			
KneeFlex Angle (^o^)	10.1 ± 7.8	6.0 ± 6.4	**< 0.001**
AnkleFlex Angle (^o^)	7.0 ± 3.9	4.8 ± 3.6	**< 0.001**

Data expressed as mean ± SD

Whereas for the lower limb parameters, significantly higher flexion angles are observed at the knee and ankle in patients with VCF than in the asymptomatic controls (*p* < 0.001). The mean KA in VCF patients and controls were 10.1^o^ ± 7.8^o^ and 6.0^o^ ± 6.4^o^, respectively. Similarly, the mean AA in VCF patients and controls were 7.0^o^ ± 3.9^o^ and 4.8^o^ ± 3.6^o^, respectively.

### Relationship between global sagittal alignment with clinical outcomes

The whole-body sagittal alignment was found to vary in patients with VCF with the associated compensation mechanism from spine to lower limb (Table 3). When analyzing patients with VCFs alone, the changes in KA and AA significantly correlated with the number of VCF (*p* < 0.01). Although both TPA and SVA significantly correlated with spinal, pelvic, and lower-limb alignment, a stronger tendency was observed in TPA when compared with SVA. Moreover, TPA was also found to be significantly correlated with the number of VCFs and SF-12 (Table 4).

**Table 3:** Correlation Coefficient between Radiographic Parameters in Patients with VCF

	TK	LL	PT	PI	SVA	TPA	KA	AA
TK	/	**0.620**^**b**^	0.019	**0.196**^**a**^	−0.040	− 0.054	0.010	0.085
LL	**0.620**^**b**^	/	−0.161	**0.393**^**b**^	**−0.277**^**b**^	**−0.361**^**b**^	**− 0.268**^**b**^	−0.129
PT	0.019	−0.161	/	**0.574**^**b**^	**0.193**^**a**^	**0.877**^**b**^	**0.356**^**b**^	**0.352**^**b**^
PI	**0.196**^**a**^	**0.393**^**b**^	**0.574**^**b**^	/	0.077	**0.493**^**b**^	0.073	0.084
SVA	−0.040	**−0.277**^**b**^	**0.193**^**a**^	0.077	/	**0.408**^**b**^	**0.250**^**b**^	0.065
TPA	−0.054	**− 0.361**^**b**^	**0.877**^**b**^	**0.493**^**b**^	**0.408**^**b**^	/	**0.489**^**b**^	**0.356**^**b**^
KA	0.010	**−0.269**^**b**^	**0.356**^**b**^	0.073	**0.250**^**b**^	**0.489**^**b**^	/	**0.849**^**b**^
AA	0.085	−0.129	**0.352**^**b**^	0.084	0.065	**0.356**^**b**^	**0.849**^**b**^	/

*TK* thoracic kyphosis, *LL* lumbar lordosis, *PT* pelvic tilt, *PI* pelvic incidence, *SVA* sagittal vertical axis, *TPA* T1 pelvic angle, *KA* kneeflex angle, *AA* ankleflex angle

^a^ Correlation significance at the 0.05 level

^b^ Correlation significance at the 0.01 level

**Table 4:** Correlation of whole-body sagittal parameters between different parameters in patients with vertebral fracture (VCF) (*n* = 142)

	No. of VCF	SF12 (PCS)	ODI	VAS
**KA**	**0.293**^**b**^	−0.123	0.730	−0.012
**AA**	**0.254**^**b**^	−0.137	0.057	−0.011
**SVA**	0.125	−0.074	0.106	0.058
**TPA**	**0.431**^**b**^	**−0.196**^**a**^	0.163	0.082

*SF-12* short form 12, *PCS* The physical component summary, *ODI* Oswestry disability index, *VAS* visual analogue scale, *KA* kneeflex angle, *AA* ankleflex angle, *SVA* sagittal vertical axis, *TPA* T1 pelvic angle

^a^ Correlation significance at the 0.05 level

^**b**^ Correlation significance at the 0.01 level

## Discussion

This study is the first to illustrate the abnormal lower limb alignment exhibited in patients with VCF. Patients with VCF have significantly increased KA and AA when compared with asymptomatic controls. This provides evidence on the contribution of the lower limb to the global compensation after insufficiency fracture of the spine occurs. Patients with VCF have an overall worse global sagittal alignment and decreased quality of life. The changes in global sagittal alignment of VCF patients also imply worse quality of life and more severe VCF.

Traditionally, vertebral collapse can lead to a structural kyphotic deformity of the spine [14, 15]. With this localized kyphotic change of the sagittal balance, our body compensates globally, with pelvic rotation, hip extension, knee flexion and ankle dorsiflexion [6], in order to maintain the optimal alignment of the spine and horizontal gaze [16, 17]. The development of EOS whole body biplanar X-ray provides a tool for better understanding of the compensatory mechanisms, which is previously difficult to assess using traditional radiographs [18, 19]. To ensure a proper treatment or rehabilitation regime is given, it would be essential to understand the sagittal compensation in VCF patients. The local kyphosis due to compression fracture is translated into the imbalance of the body alignment, causing a forward movement of the center of gravity. This predisposes to subsequent decompensation of the other segments, and ultimately leads to further vertebral collapses [9]. This may explain the overall reduced height, weight, and BMI in VCF patients, as observed in our study.

Our study demonstrated the increased spinopelvic parameters in VCF patients, including thoracic kyphosis (TK), pelvic tilt (PT) and pelvic incidence (PI). Fechtenbaum et al. also documented higher thoracic kyphosis in osteoporotic patients with VCF [7]. The global parameters, including SVA and TPA, are higher in VCF patients, signifying a global forward shift of the balance. This finding further correlates with our previous study on global sagittal compensation [8]. We documented and quantified the lower limb compensation in VCF patients, with an increased knee flexion and ankle dorsiflexion. Waters et al. documented a significant increase in energy expenditure when a person walks with a knee flexion gait [20]. With the global compensation of the body, despite the balance and the gait is compensated, a higher energy expenditure is resulted. In the long run, this can lead to chronic back pain, easy falls, or secondary osteoarthritis of the knees.

By understanding the lower limb compensation after VCF occurs, clinicians and therapists can better evaluate the severity of VCF and its effect on QoL by observing the posture and lower limb compensation of the patient, which helps formulate patient-specific rehabilitation plan to maximize its effectiveness. Surgeons can also better evaluate the optimal spinal alignment during surgical planning for spinal instrumentation, considering the alignment of the lower limb, to formulate the suitable degree of correction for best clinical function.

In addition, we have also documented a significant correlation between the number of VCFs with global sagittal alignment, in terms of TPA, as well as lower limb alignments in terms of KA and AA. The more VCFs the patient has, the more drastic the compensatory deformity over the whole body as well as the lower limb. This stress the importance of timely diagnosis of VCF with early relevant treatment and rehabilitation to prevent the occurrence of multiple vertebral collapses.

However, there remains some limitations that would need to be addressed in the current study. This study has the limitations of being a cross-sectional study. Hence, the temporal relationship between VCF and global sagittal alignments cannot be addressed. Although our study has provided an important indication of lower limb malalignment in patients with VCFs, the compensatory mechanism of the body would need a longitudinal follow-up study to demonstrate the changes after an acute collapse of the vertebral body. Associated degenerative condition, including lumbar spinal stenosis or early osteoarthritis of the hips and knees, might be confounders between the two groups, and might be further investigated by magnetic resonance imaging in future studies. In addition, the relationship of body musculature toward sagittal balance was not investigated. As sarcopenia is one of the associated conditions in patients with VCFs, its effect on sagittal alignment warrants further research and investigation.

## Conclusion

In conclusion, patients with VCFs had a generally worsen global sagittal alignment and decreased quality of life when compared with age-matched individuals. Our current study is the first to demonstrate a poor lower limb alignment, in terms of knee-flex angle (KA) and ankle-flex angle (AA), in patients with VCF. VCF patients are found to have high thoracic kyphosis (TK), pelvic tilt (PT) and pelvic incidence (PI). T1 pelvic angle (TPA), a global sagittal balance parameter, correlates with multiple local sagittal parameters of the spinopelvis and lower limb. The change in lower limb alignments was found to be strongly affected by the number of VCF in these patients. Based on our current result, there is an increased importance on the need to provide critical attention or rehabilitation strategy on the lower limb in patients with VCFs.

## Data Availability

The datasets generated and/or analyzed during the current study are not publicly available due to the privacy and sensitivity of the patients involved, but are available from the corresponding author on reasonable request.

## Abbreviations

AA: Ankleflex angle
BMI: Body mass index
GSA: Global sagittal aligments
ICC: Intra-correlation coefficients
KA: Kneeflex angle
LL: Lumbar lordosis
ODI: Oswestry disability index
PI: Pelvic incidence
PT: Pelvic tilt
SF-12: Short form 12
SVA: Sagittal vertical axis
TK: Thoracic kyphosis
TPA: T1 pelvic angle
VAS: Visual analogue scale
VCF: Vertebral compression fracture
